# Effectiveness of a volunteer befriending programme for patients with schizophrenia: randomised controlled trial

**DOI:** 10.1192/bjp.2019.42

**Published:** 2020-09

**Authors:** Stefan Priebe, Agnes Chevalier, Thomas Hamborg, Eoin Golden, Michael King, Nancy Pistrang

**Affiliations:** 1 Professor, Unit for Social and Community Psychiatry (WHO Collaborating Centre for Mental Health Services Development), Queen Mary University of London, UK; 2 Trial Manager, Unit for Social and Community Psychiatry (WHO Collaborating Centre for Mental Health Services Development), Queen Mary University of London, UK; 3 Statistician, Pragmatic Clinical Trials Unit, Centre for Primary Care and Public Health, Queen Mary University of London, UK; 4 Volunteer Coordinator, Unit for Social and Community Psychiatry (WHO Collaborating Centre for Mental Health Services Development), Queen Mary University of London, UK; 5 Professor, Division of Psychiatry, Faculty of Brain Sciences, University College London, UK; 6 Emeritus Professor, Department of Clinical, Educational and Health Psychology, University College London, UK

**Keywords:** Social isolation, volunteering, psychosis, social contacts

## Abstract

**Background:**

Befriending by volunteers has the potential to reduce the frequent social isolation of patients with schizophrenia and thus improve health outcomes. However, trial-based evidence for its effectiveness is limited.

**Aims:**

To conduct a randomised controlled trial of befriending for patients with schizophrenia or related disorders.

**Method:**

Patients were randomised to a befriending programme for 1 year or to receive information about social activities only (trial registration: ISRCTN14021839). Outcomes were assessed masked to allocation at the end of the programme; at 12 months and at a 6-month follow-up. The primary outcome was daily time spent in activities (using the Time Use Survey (TUS)) with intention-to-treat analysis.

**Results:**

A total of 124 patients were randomised (63 intervention, 61 active control) and 92 (74%) were followed up at 1 year. In the intervention group, 49 (78%) met a volunteer at least once and 31 (49%) had more than 12 meetings. At 1 year, mean TUS scores were more than three times higher in both groups with no significant difference between them (adjusted difference 8.9, 95% CI −40.7 to 58.5, *P* = 0.72). There were no significant differences in quality of life, symptoms or self-esteem. However, patients in the intervention group had significantly more social contacts than those in the control group at the end of the 12-month period. This difference held true at the follow-up 6 months later.

**Conclusions:**

Although no difference was found on the primary outcome, the findings suggest that befriending may have a lasting effect on increasing social contacts. It may be used more widely to reduce the social isolation of patients with schizophrenia.

People with schizophrenia tend to be more socially isolated than other groups in the population.^1^^,^^2^ Social isolation in turn is linked to higher levels of symptoms, poor quality of life and worse treatment outcomes.^3^^–^^5^ Specific symptoms may contribute to this social isolation: negative symptoms can affect motivation and ability to socialise^3^^,^^6^ and positive symptoms can lead to an active avoidance of social situations.^7^ Social isolation may also be driven by social exclusion, prejudice and a tendency for others to distance themselves.^8^ In contrast to this, there are volunteers who give up their spare time to support people with schizophrenia,^9^ often in the form of ‘befriending’ delivered through a dedicated programme.^10^^–^^12^ Befriending programmes generally involve a relationship between two individuals with regular input over a pre-specified period of time that is initiated and supervised by a third party.^13^ Befriending has been suggested to have benefits for the patients and volunteers, as well as for society at large through promoting social cohesion and social capital. Despite being widespread, the effects of befriending for patients have scarcely been researched.^10^^,^^11^

Meta-analyses assessing similar interventions for physical or mental health conditions suggested positive effects on depressive symptoms,^14^ and on overall patient-reported outcomes.^15^ However, effect sizes were small and trial-based evidence was inconclusive, particularly with respect to behavioural outcomes. Only two trials were conducted with patients with severe mental illness and these were with diagnostically mixed samples. One used a matched-control design and found a positive effect of befriending on perceived social support and non-significant trends on other outcomes.^16^ The second reported increased engagement in social activities following the intervention, but also found this improvement in the control group, with no significant difference between them.^17^ Against this background, we established a befriending programme with clearly defined quality criteria, including systematic training of all volunteers and regular reminders to all participants, and conducted a randomised controlled trial (RCT, trial registration: ISRCTN14021839) of its effectiveness for socially isolated people with schizophrenia. We tested whether befriending would reduce social isolation and lead to improvements in other health and social outcomes.

## Method

### Study design and participants

We conducted a parallel groups RCT in community-based mental health services in London, UK, between August 2015 and August 2017 (full details in the published protocol).^18^ Patient-participants were recruited from 15 community services across the London boroughs of City and Hackney, Tower Hamlets and Newham. Eligible patients were aged 18–65 years; had a clinical diagnosis of schizophrenia or related disorders (ICD–10: F20–29);^19^ had been in the care of the service for at least 1 month; were not current in-patients; expressed a willingness to participate in regular befriending for a year; had sufficient command of English to converse with a volunteer; and were physically able to engage in a range of community activities. Eligible patients also had a defined level of social isolation, measured on the Time Use Survey (TUS),^20^ of spending less than 60 min per day in social or recreational activities. Exclusion criteria were having already received befriending in the past 2 years; current participation in another research study; and posing a potential risk to the volunteer because of a significant history of violence.

Patients meeting the inclusion criteria that could be established from medical records were identified from service case-loads. Clinicians obtained permission from identified patients to be approached by researchers, who met the patients and established further eligibility criteria. Written informed consent was obtained from all participants after a full explanation of study procedures.

Volunteer-participants were recruited from various sources including flyers in local community centres and universities. Eligible volunteers were 18 years or older and had sufficient command of English. Exclusion criteria were the receipt of treatment from secondary mental health services in the past year, in order to distinguish befriending from peer support; a current professional role in mental health services; and any unspent criminal convictions. Eligibility was established through an application form, interviews and a criminal records check. The initial training was for 2 full days, covering general information about the programme, symptoms of schizophrenia, responsibilities and boundaries in befriending, and resources for supervision and support.

Simple 1:1 randomisation with randomly varied block lengths of 4 and 6 was used to allocate patients to the intervention or control arms. This was done by the registered Pragmatic Clinical Trials Unit at Queen Mary University of London via a dedicated website, only accessible by an unmasked researcher who in turn informed participants.

### Procedures

The intervention was developed through mapping the policies and the practices of existing programmes, and through discussions with experts including volunteer managers, volunteers and patients.^18^

Patient-participants allocated to the intervention were contacted after randomisation to arrange an initial meeting with the volunteer coordinator to establish their interests and preferences for a volunteer. After this, the volunteer coordinator arranged and facilitated an initial ‘matching’ meeting with a volunteer selected on the basis of their preferences and availability. During this meeting both parties were provided with an activity booklet, containing options for free or inexpensive activities in the local area. Following this initial meeting the volunteer and patient were asked to meet weekly for a year and encouraged to engage in joint activities. Patients and volunteers were invited to inform the volunteer coordinator within the first month if they wanted to be ‘matched’ with someone different, which would be arranged as soon as possible depending on the pool of suitable volunteers at that time. Monthly social events including food and/or an activity (for example a picnic in a park, an art workshop) were organised by the programme to provide opportunities for different volunteers and patients to meet and interact.

Volunteers reported the occurrence, length and content of meetings to the coordinator, either via text message or over the telephone. Both parties were regularly reminded of weekly meetings and could request supervision to problem-solve any emerging challenges in the relationship. Volunteer retention was encouraged and facilitated as far as possible; however, in cases where they had to drop-out, the patient was given the option of starting a relationship with a new volunteer.

Patients allocated to the active control condition were met by an unmasked researcher who provided them with an activity booklet and spoke to them about activities they might like to engage in.

Outcomes were assessed at baseline, at the end of the programme at 12 months and after a further 6 months’ follow-up. We aimed for all researchers conducting outcome assessments to remain masked to allocation and an unmasked researcher regularly reminded patient-participants to conceal their allocation status. For patients in the intervention, follow-up assessments were arranged at least 1 week following the last meeting with a volunteer. This was organised by the volunteer coordinator to ensure that measures of social outcomes did not include meetings with the volunteer.

### Outcomes

The primary outcome was average time spent engaging in activities in min per day at 12 months, measured with an adapted version of the TUS applied to the past 4 days. The TUS was developed for the general population and had previously been used with patients with schizophrenia.^20^

Secondary outcomes were: (a) social contacts defined as the number of different people met across the past 4 days and assessed using the Social Contacts Assessment;^1^ (b) observer-rated symptoms of schizophrenia assessed with both the Positive and Negative Syndrome Scale^21^ and the Clinical Assessment Interview for Negative Symptoms;^22^ (c) self-rated depressive symptoms on the Beck Depression Inventory;^23^ (d) subjective quality of life measured as the mean of the 12 satisfaction items on the Manchester Short Assessment of Quality of Life;^24^ (e) the objective social situation using the Objective Social Outcomes Index (SIX);^25^ and (f) self-esteem measured on the Self-Esteem Rating Scale- Short From.^26^

In the intervention group the number and duration of volunteer–patient meetings within the 1-year period were documented. Patients who had at least 13 meetings, representing an average of one meeting a month, were defined as ‘compliers’. This figure was set *a priori* and did not include attendance at social events without the volunteer.

### Statistical analyses

All statistical analyses were carried out in accordance with an analysis plan that was signed off prior to data extraction. The analyses were conducted using Stata (version 14.2).

The required sample size was 84. Assuming 20% attrition, a total of 106 patients needed to be recruited in order to achieve 80% power to detect a standardised effect of 0.6 at two-sided 5% significance level. This was assumed to reflect an increase of 45 min of social activities per day, that is, more than double the expected baseline average of less than 45 min. The effect of missing data was accounted for by imputing data using multiple imputations by chained equations, with a Markov chain Monte Carlo sampler burn-in of ten draws to obtain ten complete data-sets.

Each outcome was compared at 12 months between intervention and control groups using a linear regression model, adjusting for baseline score of that outcome only. For the primary outcome (TUS) the analysis on the imputed data-set was the primary analysis, with the complete case analysis as a sensitivity analysis. A further sensitivity analysis was undertaken using quantile (median) regression with robust standard error estimation to assess the influence of outlier observations.

Given that patients could drop-out of the intervention and not be lost to follow-up, a complier average causal effect (CACE) analysis was conducted.^27^ The CACE estimate was obtained by a two-stage least square instrumental variable regression, adjusting for baseline scores, under the assumption of monotonicity, exclusion restriction and the stable unit treatment value assumption.

Secondary outcomes were analysed on a complete case basis. Because the SIX is considered a ranking scale, the quantile (median) regression was used for the treatment effect estimate. The treatment effect of the count variable of number of social contacts was estimated using negative binomial regression. For those outcomes that showed significant differences between the groups at the end of the programme at 12 months, regression models, adjusting for baseline scores, were conducted to compare the groups at the 6-month follow-up.

### Ethics statement

The authors assert that all procedures contributing to this work comply with the ethical standards of the relevant national and institutional committees on human experimentation and with the Helsinki Declaration of 1975, as revised in 2008. All procedures involving participants/patients were approved by the Camden and Kings Cross Research Ethics Committee (15/LO/0674).

## Results

A total of 1245 patients were screened. Of those who were screened as eligible and met with a researcher, 55% consented to participate in the study. At this stage, a number of patient-participants were excluded because they already engaged in social activities for 60 or more min per day, decided to withdraw prior to randomisation or had provided invalid consent. This resulted in a sample of 124 patients, with 63 randomised to the intervention and 61 to the active control. After 1-year, data for analysis were obtained from 46 patients in each group. Researchers became unmasked during two of the interviews at 12 months, once before and once after the assessment of the primary outcome. The CONSORT flow diagram is available in supplementary Fig. 1, available at https://doi.org/10.1192/bjp.2019.42.

Baseline characteristics of patients are shown in Table 1. Patients were largely men with an average length of illness of 14.5 years and of varied ethnic origin.

**Table 1:** Baseline characteristics

	Intervention group (*n* = 63)	Control group (*n* = 61)
Age, years: mean (s.d.)	43.4 (10.7)	41.3 (10.0)
Gender, women: *n* (%)	22.0 (35.5)	21 (34.4)
Ethnic origin, *n* (%)		
White	10 (15.9)	9 (14.8)
Arab	2 (3.2)	0 (0)
White other	1 (1.6)	2 (3.3)
Black Caribbean	13 (20.6)	9 (14.8)
Black African	14 (22.2)	10 (16.4)
Black other	5 (7.9)	2 (3.3)
Indian/Pakistani	0 (0)	5 (8.2)
Bangladeshi	12 (19.0)	11 (18.0)
Asian other	1 (1.6)	4 (6.6)
Mixed/multiple ethnic groups	1 (1.6)	2 (3.3)
Other unspecified	4 (6.3)	7 (11.5)
Years since diagnosed, mean (s.d.)	14.8 (10.3)	14.2 (9.6)
Age at leaving full-time education, years: mean (s.d.)	18.5 (5.4)	18.6 (5.2)
Employment status, *n* (%)		
Paid employment	2 (3.2)	1 (1.6)
Training/education	1 (1.6)	3 (4.9)
Unemployed	57 (90.5)	56 (91.8)
Retired	1 (1.6)	0 (0)
Other	2 (3.2)	1 (1.6)
Receiving benefits, yes: *n* (%)^a^	58 (93.5)	51 (85.0)
Participants with children, *n* (%)^a^	21 (33.9)	22 (36.6)
Living situation, *n* (%)^b^		
Live alone	39 (61.9)	39 (65.0)
With partner and/or children	12 (19.0)	8 (13.3)
With parents	10 (15.9)	5 (8.3)
Other	2 (3.2)	8 (13.3)

a. Data not obtained for one control and one intervention participant.

b. Data not obtained for one control participant.

Fifty-one volunteers were recruited and met with at least one patient. Most were women (73%, *n* = 37), from a white ethnic group (65%, *n* = 33) and with previous volunteering experience (71%, *n* = 36). The average age of volunteers was 27.8 (s.d. = 8.9) with a modal age of 21 years. Most volunteers were in full-time or part-time employment (59%, *n* = 30), with some in full-time education (22%, *n* = 11) or unemployed (10%, *n* = 5) and only one had retired.

Of the 63 patients allocated to the intervention, 14 never met a volunteer (22%). For five patients further contact at that stage revealed that they did not meet all eligibility criteria (one had previous befriending experience, one had a history of violence, three lost capacity to consent to the intervention) and nine were no longer interested once the reality of being matched with a volunteer became apparent.

**Fig. 1:**
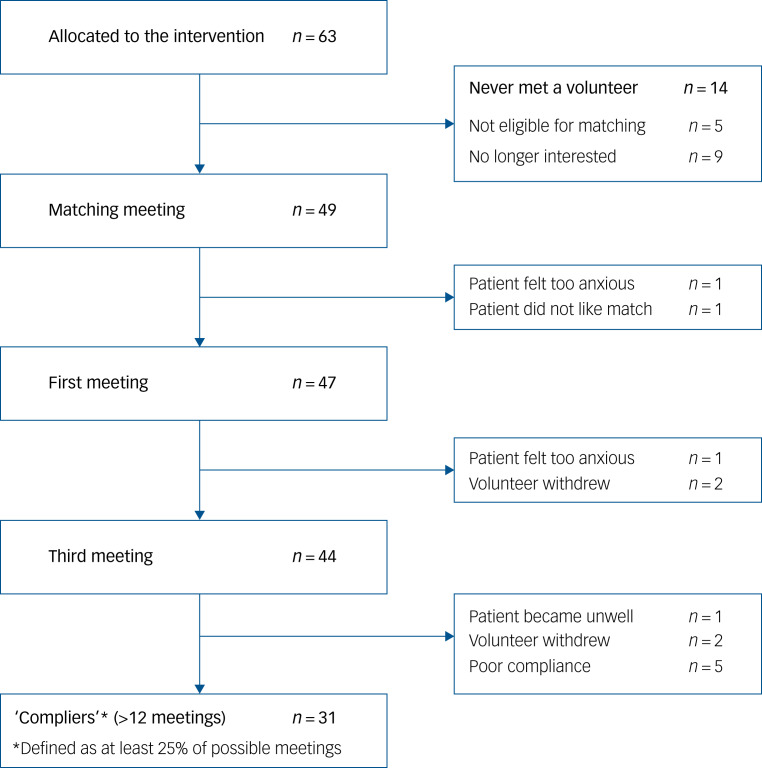
Befriending programme flow diagram.

Among those with an initial ‘matching’ meeting (*n* = 49) implementation of the programme was limited. Only half of the intended sample achieved the pre-defined threshold of at least 13 meetings (Fig. 1). For those who had at least one meeting, the median number of meetings across the year was 14 (range 1–42) with a mean duration of 90 min. Overall, 15 social events were organised and attended by a mean of six patients (range 3–9). Five patients were re-matched and thus had two volunteers over the course of the year. For four of these patients this was because their volunteer could no longer meet their commitment and had to drop-out of the programme. For the other patient their original volunteer was a woman and they expressed a strong preference for a befriender of the same gender.

In the intention-to-treat analysis, there was no significant difference between the intervention and active control groups on the primary outcome, that is, the time patients spent in activities at 12 months (adjusted difference 8.9, 95% CI −40.7 to 58.5, *P* = 0.72). Patients in the intervention group increased their activity from 20 to 81 min per day in the primary analysis with imputed data, which represents a larger difference than the one that was considered to be clinically meaningful for the sample size calculation. However, a similar increase (from 17 to 70 min) was found in the control group (Table 2.) The analysis of complete cases and analyses using quantile regression produced similar results. The CACE analysis exploring the association between compliance in the intervention arm and the primary outcome was non-significant (adjusted difference  9.2, 95% CI −68.9 to 87.2, *P* = 0.82).

**Table 2:** Primary outcome analysis

	Intervention	Control	Treatment effect (95% CI)^a^	*P*	Intervention, *n*	Control, *n*
Baseline time spent in activities (TUS), mean (s.d.)	20.03 (21.05)	17.41 (19.99)			63	61
Primary analysis						
TUS at 12 months, mean (s.e.)	81.29 (18.43)	70.33 (15.72)	8.90 (−40.69 to 58.50)	0.720	63	61
Sensitivity analyses						
Complete case at 12 months, mean (s.d.)	72.80 (120.51)	66.66 (108.42)	5.39 (−41.77 to 52.56)	0.821	46	46
Quantile regression at 12 months, mean (s.e)	81.29 (18.43)	70.33 (15.72)	14.63 (−25.14 to 54.39)	0.463	63	61
Complete case quantile regression, median (IQR)	30.00 (60.00)	21.25 (76.25)	15.00 (−12.95 to 42.95)	0.289	46	46

IQR, interquartile range.

a. Adjusted for baseline levels of Time Use Survey (TUS).

Data for secondary outcomes were available for 69–74% of patients (Table 3). For symptoms, quality of life and self-esteem there were no significant differences between the groups. However, patients in the intervention group had significantly more social contacts (adjusted difference 0.52, 95% CI 0.04–0.99, *P* = 0.03) and more favourable SIX scores (adjusted difference 2.45, 95% CI 1.06–5.67, *P* = 0.04). Since the SIX contains one item about having met a friend in the past week (no, 0; yes, 1), we conducted a *post hoc* analysis to determine whether that item drove the improvement on the SIX. The difference on that item alone was significant (intervention group: 19/62 = 31% at baseline and 22/45 = 49% at follow-up; control group: 23/61 = 38% at baseline, and 14/46 = 30% at follow-up, *P* = 0.04). Without that item, there was no significant difference between the groups (adjusted difference 0.92, 95% CI 0.36–2.33, *P* = 0.86).

**Table 3:** Secondary outcomes

	Intervention, mean (s.d.)	Control, mean (s.d.)	Mean difference (95% CI)	Treatment effect (95% CI)^a^	Intervention, *n*	Control, *n*
Self-esteem (Self-Esteem Rating Scale)						
Baseline	88.08 (22.86)	87.79 (23.80)	0.29 (−8.04 to 8.62)		62	61
12 months	94.14 (20.89)	91.44 (26.55)	2.69 (−7.48 to 12.87)	3.14 (−4.84 to 11.12)	44	43
PANSS general						
Baseline	28.24 (7.97)	30.36 (7.25)	−2.12 (−4.84 to 0.60)		62	61
12 months	26.58 (8.85)	28.58 (7.59)	−2.00 (−5.45 to 1.45)	−1.68 (−5.00 to 1.64)	45	45
PANSS negative symptoms						
Baseline	17.23 (7.47)	16.84 (7.11)	0.39 (−2.21 to 2.99)		62	61
12 months	16.13 (7.79)	14.67 (7.15)	1.47 (−1.67 to 4.60)	1.35 (−1.55 to 4.25)	45	45
PANSS positive symptoms						
Baseline	11.40 (4.18)	13.67 (4.83)	−2.27 (−3.88 to −0.65)		62	61
12 months	11.73 (5.09)	13.16 (6.29)	−1.42 (−3.82 to 0.974)	−0.43 (−2.71 to 1.85)	45	45
CAINS negative symptoms scale						
Baseline	26.29 (9.21)	27.98 (9.13)	−1.69 (−4.97 to 1.58)		62	61
12 months	25.36 (9.40)	27.74 (9.05)	−2.38 (−6.23 to 1.46)	−2.00 (−5.79 to 1.78)	45	46
Beck Depression Inventory-II						
Baseline	15.85 (12.60)	19.16 (13.95)	−3.31 (−8.05 to 1.43)		62	61
12 months	18.12 (13.07)	15.88 (12.56)	2.23 (−3.30 to 7.77)	3.32 (−0.45 to 7.09)	43	42
Subjective quality of life (MANSA)						
Baseline	4.26 (0.92)	3.98 (1.10)	0.27 (−0.09 to 0.64)		62	61
12 months	4.45 (1.00)	4.31 (0.83)	0.14 (−0.24 to 0.53)	−0.00 (−0.31 to 0.31)	44	46
Social contacts (people met in last 4 days, *n*)						
Baseline	1.56 (2.59)	1.33 (1.69)	0.23 (−0.56 to 1.02)		59	61
12 months	1.88 (2.00)	1.28 (1.61)	0.60 (−0.17 to 1.37)	0.52 (0.04 to 0.99)*	42	46
18 months	4.03 (6.59)	1.95 (2.89)	2.08 (−0.27 to 4.43)	0.73 (0.05 to 1.40)*	38	37
Objective Social Outcomes Index						
Baseline	2.55 (0.99)	2.61 (0.79)	−0.06 (−0.38 to 0.26)		62	59
12 months	2.86 (0.93)	2.66 (1.25)	0.20 (−0.26 to 0.67)	2.45 (1.06 to 5.67)*^b^	44	44
18 months	2.90 (0.99)	2.67 (1.24)	0.23 (−0.29 to 0.75)	3.05 (1.13 to 8.20)*^b^	39	36

a. Adjusted for baseline levels of the outcome.

b. Ordered logistic regression model estimate.

PANSS, Positive and Negative Syndrome Scale; CAINS, Clinical Assessment Interview for Negative Symptoms; MANSA, Manchester Short Assessment of Quality of Life

* *P* < 0.05.

The analyses comparing the groups at the 6-month follow-up showed that patients in the intervention group still had significantly more social contacts (adjusted difference  0.73, 95% CI 0.05–1.40, *P* = 0.04), and better scores on the SIX (adjusted difference 3.05, CI 1.13–8.20, *P* = 0.03).

**Patient perspectives**

Patients reported various positive experiences of the programme, to which they assigned a range of benefits such as feeling more understood or self-confident, and having a greater sense of belonging to society at large.

‘I feel more confident about myself. I feel like people understand me more, that there is somebody that can be there. And she always talks to me about my feelings and makes me feel better.’ (Patient 30)

‘She thought that schizophrenics were interesting people who were worthy of getting to know and being treated with respect and not patronised or looked down upon or treated as if they were kind of kiddies. That was very therapeutic.’ (Patient 131)

Negative experiences of the programme were usually found among those who dropped out, either because they felt uncomfortable with the volunteer with whom they had been matched, or because the volunteer was unreliable and had themselves, dropped out.

‘I feel sometimes embarrassed because he's too young, he is younger than me and I want somebody… like at the most who is five years younger than me, four or five.’ (Patient 4)

‘I felt like I was pushing her to come without her being willing to come and meet me and go to the library and stuff like that. I feel a bit bad about that because for me it felt like she didn't want to be here. Like she was being forced to come and meet with me.’ (Patient 85)

## Discussion

In this trial of a befriending programme for patients with schizophrenia, time spent in activities increased substantially in both arms but with no differential benefit for befriending. In terms of secondary outcomes, patients in the befriending programme had significantly more social contacts after 1 year – as reflected across two measures – and this difference held true 6 months after the end of the programme. This advantage was not associated with significant improvements in symptoms, quality of life or self-esteem. Both patients and volunteers engaged with the programme variably, and overall there were fewer meetings between patients and befrienders than envisaged. About a quarter of patients either never met a volunteer or did not proceed beyond the initial matching stage, and practically none had as many meetings as planned in the programme design.

### Strength and limitations

This is the largest known RCT of befriending in a diagnostically homogeneous group of patients with schizophrenia.^16^^,^^17^ The befriending programme was carefully designed and implemented including both selection interviews and training for volunteers, frequent reminders, access to supervision and the offer of regular social events. A range of observer-rated and self-rated outcomes were assessed, and the programme organisers ensured that patients' accounts of social activities at the end of the programme were not influenced by meetings with the volunteers themselves. Finally, the positive findings on increased social contacts were found on two measures and at two points of time (i.e. at the end of the programme and 6 months later).

The study also has a number of weaknesses. The chosen primary outcome of time spent in social activities showed a more than threefold increase in both groups. Levels of time spent in social activities appear to have been lower at baseline and higher at follow-up compared with those reported in a trial with patients in early intervention services, in any group and at any point of time.^28^ This might raise questions about the validity of the measurement of this outcome. There was a protocol violation for five patients who had to be excluded from the intervention following randomisation. The follow-up rate of 74% is slightly lower than the average in trials on psychosocial interventions in patients with schizophrenia, although similar in both groups.^29^ Moreover, the trial may have been influenced by specific contextual factors affecting the volunteers, such as the predominant recruitment through advertising in universities. This resulted in many of the volunteers being in their early twenties and much younger than the patients they befriended. Finally, the trial focused on gains that patients had achieved through befriending, whereas potential benefits for volunteers and for the wider community were not assessed.

### Comparison with the literature

A meta-analysis across physical and mental health conditions^15^ and a quasi-experimental study in the USA^16^ suggested a positive effect of befriending on patient-reported outcomes. Our RCT did not identify such benefits (i.e. depressive symptoms, subjective quality of life and self-esteem). Another departure from previous studies is a group difference on a behavioural outcome. The higher number of social contacts in the intervention group potentially reflects a reduction in social isolation and a gain not previously demonstrated in other studies. At baseline, patients had contacts with an average of one to two different people over a period of 4 days. Befriending increased this by an average of 0.5 people, albeit with considerable variation. Whether this increase represents a relevant change in the social life of patients with psychosis is difficult to judge, but should be encouraging given that the benefit was upheld 6 months later.

Our primary result is similar to an Irish trial with patients with severe mental illnesses^17^ in which marked improvements were found in both intervention and control groups. In that trial, both groups received financial support for social activities – in addition to the befriending provided to the intervention group – so the authors argued that the improvement across groups was because of the provision of financial support. In our trial, the control group only received information about social activities. This information, discussed on one occasion, is unlikely to explain a more than threefold increase in time spent in social activities 1 year later. One can only speculate as to whether this was a general Hawthorne effect of participating in a research study, or whether patients' accounts were influenced by a social desirability bias, motivating patients in both groups to inflate their reports at follow-up, whereas at baseline, reports of spending too much time in activities would have made them ineligible for the trial. Retrieving data on social contacts that included counting face-to-face meetings with different people or answering one dichotomous question about whether they had met a friend may have been less sensitive to such bias.

The considerable variability in the uptake and use of the programme may be explained by a recent survey of out-patients with psychotic and affective disorders.^30^ Patients expressed a wide range of preferences for befriending in terms of frequency and content of meetings (i.e. talking or engaging in joint activities), the duration of the programme and the background of the volunteer. Similarly, volunteers also vary in their motivations, expectations and aims for befriending.^10^^,^^11^^,^^31^ As they are not salaried staff, they cannot necessarily be expected to follow instructions and may not feel obliged to adhere to a fixed programme. Finally, it is difficult to judge the impact of large differences in age and background, present in many of the patient–volunteer dyads, which may have hindered the development of close social connections. Overall, one may conclude that a one-size-fits-all befriending programme is likely to have limited adherence.

### Implications for research and practice

These findings have implications for both practice and research. In practice, patients and volunteers should be offered programmes with sufficient flexibility to accommodate their varying initial preferences and changes in preferences over time. This may be in regards to the duration of the relationship, the frequency of meetings, the personal motivations of the volunteer etc.

For research, the question arises as to whether RCTs are the most appropriate method for evaluating befriending programmes. Trials focus on gains achieved at the end of an intervention period. Yet, in the case of befriending relationships over 12 months, there may be experiences during that period that are not reflected in changed outcome criteria at the 1-year point, but are still important for patients, perhaps making overall participation worthwhile. In this trial we obtained data on the uptake, use and outcomes of befriending that are of interest, without considering outcomes in a control group. A first task for future research may be to collect such data from a large number of befriending programmes currently in practice. This may provide important information on the characteristics of patients and volunteers in befriending programmes, how often and for how long they meet; the characteristics of patients, volunteers and programmes that predict more enjoyable and longer lasting relationships; the nature of the experiences of patients and volunteers; and changes in outcome criteria, both self-report and behavioural. Given that many programmes are run by voluntary organisations with limited capacity for data documentation, this research might be difficult to conduct. Yet, collecting such data on a large scale may help to better understand the potentials and limitations of befriending. This is important as reviews and large surveys suggest that volunteers for befriending programmes with people with severe mental illnesses can be recruited from groups with very different characteristics and that there is a large pool of potential volunteers who could provide valuable input to patients.^10^^,^^11^^,^^31^ Supporting their commitment and activities with the best possible evidence may be seen as a priority for research in public mental health.

## Supplementary material

For supplementary material accompanying this paper visit https://doi.org/10.1192/bjp.2019.42. click here to view supplementary material
