# Supplementary figures and images for: Effectiveness of a volunteer befriending programme for patients with schizophrenia: randomised controlled trial
Source: Br J Psychiatry. 2020 Sep;217(3):477–83. doi: 10.1192/bjp.2019.42 (PMC7116000; doi:10.1192/bjp.2019.42)

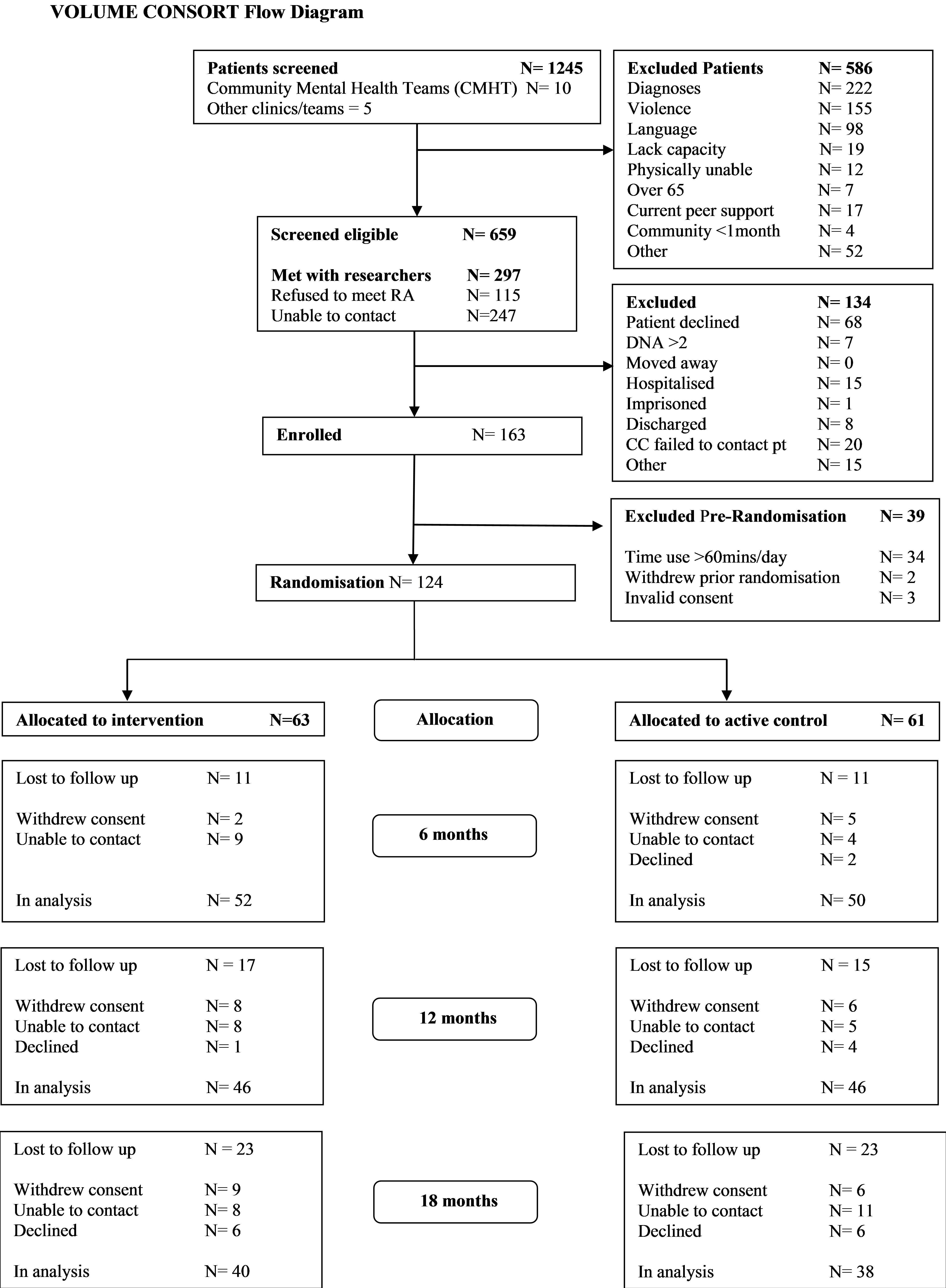

Supplement: Supplementary file 1 [file S0007125019000424sup.zip › S0007125019000424sup001.tif]
